# EspE3 plays a role in the pathogenicity of avian pathogenic *Escherichia coli*

**DOI:** 10.1186/s13567-023-01202-9

**Published:** 2023-08-29

**Authors:** Qianwen Li, Zhao Qi, Dandan Fu, Bo Tang, Xiangjun Song, Ying Shao, Jian Tu, Kezong Qi

**Affiliations:** 1 Anhui Province Key Laboratory of Veterinary and Disease Control, College of Animal Science and Technology, Anhui Agricultural University, Hefei, 230036 China; 2 Anhui Province Engineering Laboratory for Animal Food Quality and Biosafety, College of Animal Science and Technology, Anhui Agricultural University, Hefei, 230036 China; 3 Institute of Veterinary Immunology & Engineering, Jiangsu Academy of Agricultural Sciences, Nanjing, China; 4 National Research Center of Veterinary Bioproduct Engineering and Technology, Jiangsu Academy of Agricultural Science, Nanjing, China; 5 Guotai (Taizhou) Veterinary Biotechnology Innovation Center, Jiangsu Academy of Agricultural Science, Nanjing, China

**Keywords:** APEC, ETT2, effector, EspE3, pathogenicity

## Abstract

APEC encodes multiple virulence factors that have complex pathogenic mechanisms. In this study, we report a virulence factor named EspE3, which can be secreted from APEC. This protein was predicted to have a leucine-rich repeat domain (LRR) and may have a similar function to IpaH class effectors of the type III secretion system (T3SS). For further exploration, the regulatory correlation between the *espE3* and ETT2 genes in APEC was analysed. We then assessed the pathogenicity of EspE3, detected it in APEC secretion proteins and screened the proteins of EspE3 that interact with chicken trachea epithelial cells. This study provides data on a new virulence factor for further exploring the pathogenic mechanism of APEC.

**Supplementary Information:**

The online version contains supplementary material available at 10.1186/s13567-023-01202-9.

## Introduction

Gram-negative bacteria are one of the main pathogens of mammals, among which pathogenic *Escherichia coli* (*E. coli*) is a very important group. Previous studies focused on enteropathogenic *E. coli* (EPEC). As a pathogen with increasing pathogenicity that affects a large range of hosts, extraintestinal pathogenic *E. coli* (ExPEC) has a variety of pathogenic mechanisms that are not yet clear. Avian pathogenic *E. coli* (APEC) is a typical ExPEC that can cause systemic infection in hosts. A variety of studies have shown that APEC can infect poultry through the respiratory tract, bypass the immune barrier of the host, and cause a variety of diseases, such as air sacculitis, pericarditis, perihepatic inflammation, and bacteraemia [1–4]. Many kinds of virulence factors and secretion systems assist in APEC infection and colonization. The pathogenic mechanisms of many virulence factors in APEC are still not clear.

*E.coli* type III secretion system 2 (ETT2), encoded in the genome of APEC [5, 6], participates in the regulation of a variety of pathogenic phenotypes and in the pathogenicity characterization of APEC, ETT2 has been described as an important bacterial virulence gene cluster. APEC-ETT2 encodes a variety of transcriptional regulators, such as YqeI [7], EivF [8], YgeH [9] and EtrA [10], that regulate the pathogenicity of APEC. At the same time, APEC-ETT2 also encodes ATPase EivC [11] and the molecular chaperone YgeG [12] to contribute to pathogenicity. As a secretion system, ETT2 was thought to secrete effectors. In the draft genome sequencing of the APEC clinical isolate APEC81 (AE81), we focused on a gene that encodes a protein with a similar domain to SlrP. SlrP is a protein with E3 ubiquitin ligase activity that is translocated by *Salmonella enterica* serovar Typhimurium into eukaryotic host cells through a type III secretion system. We speculate that the gene may have a similar function to *slrP*. In this study, we report the pathogenicity of the gene we named *espE3* (*Escherichia coli* secretion protein E3s ubiquitin ligase), which can be secreted from APEC-ETT2.

## Materials and methods

### Bacterial strains and culture conditions

The *E. coli* strains utilized for this study were AE81 and the AE81 mutant strain with *yqeH* deletion (Δ*yqeH*) [9], *ygeG* deletion (Δ*ygeG*) [12], and ETT2 cluster deletion (ΔETT2) [13], all constructed and stored by the Anhui Province Key Laboratory of Veterinary Pathobiology and Disease Control (Table 1). The *espE3* gene deletion strain (Δ*espE3*) and complementary strain (C*espE3*) in AE81 were constructed in this study. The APEC strains were inoculated on Luria Bertani (LB) agar medium from the cryotube and then cultured in LB to the logarithmic phase.

**Table 1:** **Strains and plasmids used in this study.**

Strain or plasmid	Genotype or comments	Source
APEC81(AE81)	Wild type strain, a clinical isolate from ducks, nonpigmented	[16]
AE81-Δ*yqeH*	*yqeH* gene was deleted from AE81 genome	This study
AE81-Δ*ygeG*	*ygeG* gene was deleted from AE81 genome	This study
AE81-ΔETT2	ETT2 gene was deleted from AE81 genome	This study
AE81-Δ*espE3*	*espE3* gene was deleted from AE81 genome	This study
AE81-C*espE3*	*espE3* gene was complemented into AE81-Δ*espE3*	This study
DH5α	Strains used for plasmid cloning	Laboratory preservation
BL21(DE3)	BL21 (DE3) Competent Cells, which is used to efficiently express genes cloned in expression vectors containing phage T7 promoters (such as pET series)	Laboratory preservation
Plasmids		
pKD46	Expression lambda Red-recombinase *Exo*, *Bet* and *Gam*, temperature sensitive, ampicillin resistant	Laboratory preservation
pKD3	Amplify chloramphenicol resistance fragment Cat	Laboratory preservation
pCP20	FLP + λcI857 + λpRRep (Ts, Temperature-sensitive plasmids, ampicillin resistance and chloramphenicol resistance	Laboratory preservation
pSTV28	Low copy number cloning vector, chloramphenicol resistance	Laboratory preservation
pET-32a( +)	*E. coli* protein expression vector, His-tagged, ampicillin resistant	Laboratory preservation
pET-32a-*espE3*	His-tagged EspE3 prokaryotic expression vector, ampicillin resistance	This study
pGEX-6P-1	E. *coli* protein expression vector, containing GST tag, ampicillin resistance	Laboratory preservation
pGEX-6P-1-EspE3	GST-tagged EspE3 prokaryotic expression vector, ampicillin resistance	This study

### Gene amplification and protein model analysis of APEC81-EspE3

As a clinically isolated strain of APEC, AE81 was used to detect whether there was any effector secreted by or that assisted in secretion by ETT2. The genome of AE81 was obtained and analysed by our laboratory in the early stage [14], and the gene sequence of *espE3* was obtained. Then, we carried out sequence alignment and protein prediction on this gene. Primers for *espE3* were designed (Additional file 1), and the target fragment was amplified in AE81 and sent to Sangon Biotech Co., Ltd. for sequencing (Songjiang District, Shanghai, China). The gene nucleotide sequence was obtained, and sequence alignment was performed using the BLAST Nucleotide program. The gene sequence was predicted by Softberry FGENESB software, the amino acid sequence of gene translation was obtained, the protein model was established in SWISS-MODEL, and the protein domain was analysed by SMART. Amino acid sequence alignment was performed by BLAST-Protein.

### Detection of *espE3* in APEC clinical isolates

In this study, specific detection primers were designed according to the gene sequences of *espE3* in AE81 (Additional file 1). Using 107 APEC strains isolated from diseased chickens and ducks in Anhui Province and Jiangsu Province of China from 2015 to 2019 and stored at Anhui Province Key Laboratory of Veterinary Pathobiology and Disease Control, *espE3* was detected by PCR, and the distribution in clinical isolates was calculated. The PCR program was implemented according to the 2 × Taq Master Mix (Dye Plus) (Vazyme, Qixia District, Nanjing City, Jiangsu Province, China) manufacturer’s instructions.

### RNA isolation and RT‒PCR

In this study, we used *ygeG* [12] and *yqeH* [9] coded in the ETT2 cluster, which was shown to affect the virulence of APEC in our previous studies, as a representative to test whether the ETT2 genes affect the transcription of *espE3*. The effects of ETT2, *ygeG* and *yqeH* on the transcription level of the *espE3* gene were detected by fluorescence quantitative PCR as described previously [15]. The transcription of *espE3* in each strain at different growth stages (OD_600_ = 0.8, 1.3, 1.7) was detected. ΔETT2, Δy*qeH* and Δ*ygeG* of AE81 were generated using the standard λ Red recombinase system with the pKD46 and pKD3 template plasmids and the pCP20 FLP recombinase plasmid [9, 12, 13]. AE81, ΔETT2, Δy*qeH* and Δ*ygeG* were cultured at 37 °C, and total RNA was extracted using the Bacteria RNA Extraction Kit (Vazyme, Qixia District, Nanjing City, Jiangsu Province, China) according to the manufacturer’s instructions. The RNA was used for cDNA synthesis and further microarray analysis. SYBR green detection was used for quantitative RT‒PCR. Each reaction was conducted in triplicate two-step multiplex RT‒PCR assays with 2 × AceQ qPCR SYBR Green Master Mix (Vazyme, Qixia District, Nanjing City, Jiangsu Province, China). APEC 16S DNA served as an internal reference gene. The relative expression levels were measured using the 2^−ΔΔCT^ method.

### Construction of the *espE3* deletion and complementary strains.

*espE3* gene deletion from the AE81 chromosome was performed using the standard λ Red recombinase system with pKD46, pKD3 template plasmid and pCP20 FLP recombinase plasmid as previously described [7, 16]. First, the chloramphenicol (cm) resistance cassette was obtained from pkD3, the *espE3* gene was replaced by the cm cassette, and Δ*espE3*::cm was constructed. Subsequently, Δ*espE3* was obtained using FLP recombinase expressed by pCP20. The mutation was confirmed by PCR and DNA sequencing.

To complement the mutant, we created a complementation plasmid of pSTV28-*espE3* by using the primer pair *espE3* Co-F and *espE3* Co-R (Additional file 1) to amplify the *espE3* gene (including its putative promoter) and subcloned it into the pSTV28 plasmid. The recombinant plasmid was transformed into the mutant Δ*espE3* strain to construct the complemented C*espE3* strain.

### Determination of bacterial biological characteristics

For determination of bacterial growth ability, the growth curve of the AE81, Δ*espE3* and C*espE3* strains was assessed in LB broth as described in a previous study [15]. Briefly, overnight cultures were prepared in LB broth at 37 °C with shaking at 180 rpm. The next day, the optical density (OD) of each strain was estimated by spectrophotometry. Cultures were diluted, and the OD was again estimated by spectrophotometry to an approximate initial concentration of OD_600_ = 0.01 at the starting time point (0 h). The bacterial cultures were incubated at 37 °C with shaking at 180 rpm, three duplicate samples were taken at each time, and the OD_600_ was monitored at 2 h intervals.

For detection of mobility, the strains were inoculated into LB liquid medium and cultured until the logarithmic growth period. The bacterial culture medium was centrifuged for 3 min, the bacteria were resuspended in PBS for precipitation, and the bacteria were washed twice. Then, 2 µL of the bacterial suspension (OD_600_ = 2.0) was added dropwise onto the semisolid medium (2.0 g tryptone, 1.0 g sodium chloride, 1.6 g glucose, 0.5 g agar powder, 200 mL ultra-pure water) and observed after standing at 37 ℃ for 8 h. This test was repeated 3 times.

For detection of biofilm formation, the strains were incubated statically at 37 °C for 15 h in LB medium. Subsequently, the media was withdrawn, and the tubes were rinsed with water. Next, biofilms were fixed with 5 mL 100% methanol for 5 min. After removing the methanol, 5 mL 0.1% crystal violet solution was added and the samples were incubated for 15 min at room temperature. The solution was then removed, the tubes were washed with water, and the polystyrene tubes were air-dried without a lid for 30 min. Finally, the CV remaining in the tube was dissolved in 33% glacial acetic acid, 150 µL was added to the wells of 96-well microtiter plates, and the plate was read in single wavelength mode at 595 nm using a MicroELISA automatic reader. This experiment was independently repeated three times.

For the detection of serum sensitivity, the strains were cultured to the log growth phase in LB medium, and the culture solution was collected, centrifuged and washed with PBS. The number of bacteria was adjusted to 1 × 10^8^ CFU/mL. Ten microlitres of bacterial solution was added to 190 μL of different concentrations of serum (10%, 30%, 50%, 75%, 100%) or heat-inactivated serum (serum inactivated at 56 ℃ for 40 min); the samples were then mixed well, cultured at 37 ℃ for 30 min, and then diluted to obtain the treated bacterial solution gradient. Ten microlitres of bacterial suspension was then collected for counting using the hanging plate method, and three duplicate samples were taken each time.

For detection of adhesion and invasion, the strains were cultured to the log growth phase, centrifuged, washed with sterile PBS, and resuspended in culture medium. Chicken tracheal mucosal epithelial cells (CTE) were inoculated into a 6-well cell culture plate with epithelial cell complete medium (epithelial cell medium, 10% Gibco foetal bovine serum, 5% epithelial cell growth factor, 1% Penicillin‒Streptomycin Solution) (iCell Bioscience Inc., Xuhui District, Shanghai, China) and cultured overnight at 37 ℃ and 5% CO_2_. After the cells covered the bottom of the plate, the culture medium was discarded, and the cells were washed with sterile PBS. Cells were infected with a multiplicity of infection (MOI) = 100. The same volume of medium without FBS and antibiotics was used as the control and incubated at 37 ℃ for 1 h. The cell culture medium was discarded, and the cells were washed with sterile PBS. For the invasion test group, culture medium containing gentamicin (100 μg/mL gentamicin) was added, and the cells were incubated at 37 ℃ and 5% CO_2_ for 1 h. Finally, 0.5% Triton X-100 was added to lyse the cells, which were left to stand for 10 min and suspended slowly. The cells were counted in a hanging plate after dilution at 1:10^5^ with PBS. Three repetitions per assay were carried out.

### Detection of cellular inflammatory factors

CTE cells were seeded in 6-well cell culture plates and cultured overnight at 37 ℃ and 5% CO_2_. When the cells were completely confluent, the medium was discarded, and the cells were washed three times with sterile PBS. Strains were cultured to the log phase, centrifuged and washed with sterile PBS. Then, the strains were resuspended in medium and infected at an MOI = 100. The same volume of medium without FBS and antibiotics was used as a control and the samples were incubated at 37 °C for 12 h. RNA was extracted from the infected CTE and reverse transcribed into cDNA. β-Actin was used as the internal reference for CTE. Finally, three repeated RT‒qPCR experiments were performed. The cells cultured in sterile medium were used as a blank control to detect the transcription of inflammatory factors in CTE infected with AE81, Δ*espE3* and C*espE3*.

### Animal survival rate and the preparation of pathological sections

Strains were separately added to 100 mL liquid LB medium (1:100) and cultured at 37 °C until the log phase. Cells were collected and rinsed with PBS, and the number of bacteria was adjusted to OD_600_ = 1.0 (1 × 10^8^ CFU/mL). 7-day-old Roman chicks (*n* = 8) were inoculated with 1 × 10^8^ CFU (1 mL). The control group was treated with sterile PBS. Chicks were infected by tracheal injection and observed for 7 days after infection. Survival was monitored daily. After dissecting the chicks that died due to APEC and the control chicks, the pathological changes in the organs were observed, and tissue was prepared for paraffin-embedding of sections and haematoxylin and eosin (H&E) staining. Histopathological changes were observed by light microscopy.

### Detection of EspE3 in AE81 secretion proteins

We expressed EspE3 and prepared an anti-EspE3 polyclonal antibody serum to detect EspE3 of APEC. The genome of AE81 was used as a template for designing the *espE3* primers (Additional file 1). The pET-32a-*espE3* recombinant vector was constructed (Table 1). The expression plasmids were transformed into *E. coli* BL21 (DE3). The protein was expressed with IPTG induction at a final concentration of 2.5 mmol/L and purified using a His-tag Protein Purification Kit (Beyotime Biotechnology, Songjiang District, Shanghai, China) according to the manufacturer’s instructions. Protein samples were separated by SDS‒PAGE and either stained with Coomassie brilliant blue or transferred electrophoretically onto a nitrocellulose membrane. For antigenicity of the proteins (His-tagged fusion proteins), the membranes were blocked with 5% skim milk. Then, the membrane was incubated with anti-6 × His Tag mouse monoclonal antibody (1:1000) (Sangon Biotech Co., Ltd., Songjiang District, Shanghai, China), followed by washing in PBST. The membrane was incubated with a horseradish peroxidase (HRP)-conjugated goat anti-mouse IgG (H + L) antibody (1:5000) and then washed in PBST. The immunoreactive bands were visualized using the BeyoECL Plus Western blot detection system according to the manufacturer’s instructions.

For preparation of the polyclonal antibody, the purified proteins were used as antigens for the immunization of mice, and the antiserum was raised. Briefly, mice were randomly divided into two groups. Pre-immune serum was collected prior to immunization. The purified proteins were emulsified as a 1:1 (v/v) mixture with Freund's complete adjuvant. Mice were immunized subcutaneously with 160 μg of the purified proteins. Then, the mice were injected subcutaneously with 160 μg of the proteins mixed with Freund’s incomplete adjuvant every two weeks twice after the first immunization. The mice were euthanized and bled for two weeks after the last injection. Serum was collected and stored in aliquots at -80 ℃.

To verify the specificity of serum anti-EspE3 and the secretion of EspE3, positive serum was used as the primary antibody (1:500), HRP-conjugated goat anti-mouse IgG (H + L) antibody (Servicebio, Jiangxia District, Wuhan City, China) was used as the secondary antibody (1:5000), purified His-EspE3 was used as the positive control, the culture medium samples of AE81 and ΔETT2 were taken as the test samples, and Western blotting was performed.

### Glutathione-S-transferase (GST) pull-down in vitro assay

Utilizing pGEX-6P-1 as the vector and BL21 as the host bacteria, the prokaryotic expression vector pGEX-6P-1-EspE3 was constructed, and GST-EspE3 expression was measured and samples were purified using a GSH purification column. To screen the proteins that interact with EspE3 in the host cell CTE, a GST pulldown assay was performed.

Purified GST-EspE3 was concentrated and dialyzed using a Millipore ultrafiltration centrifuge tube with a pore size of 3 kDa. Western and IP cell lysates were used to obtain cell protein. In this assay, GST-EspE3 was used as bait protein, CTE proteins were used as prey protein, and negative control 1 (no bait protein), negative control 2 (no prey protein) and the interaction group (bait protein and prey protein interaction) were used as controls. The assay was performed as follows: briefly, GST-Sefinose™ Resin Binding/Wash Buffer (pH 7.3~7.5) (Sangon Biotech Co., Ltd., Songjiang District, Shanghai, China) was used to balance the GST agarose gel, 125 μg GST-EspE3 or the same volume of buffer was added, and the GST purification column was washed after incubation at 4 ℃ for 1 h. Then, 200 μg of prey protein was added to the incubated column and the samples were placed in a shaker incubator at 4 ℃ overnight. GST-Sefinose™ Resin Elution Buffer (pH 7.9~8.1) (Sangon Biotech Co., Ltd., Songjiang District, Shanghai, China) was prepared, 100 μL elution buffer was added to the column, and the column was incubated on a shaking table at 4 ℃ for 10 min. The elution buffer collected was the interacting protein solution. SDS‒PAGE of the interacting protein solution was performed, and the gel was stained with silver. Protein bands were measured with HPLC and MS detection by Bgi Genomics Co., Ltd. We used the *Gallus gallus* database of UniProt to match the sequencing data, filtered and controlled the search results, and obtained reliable protein identification results. Finally, based on the final protein identification list, GO, COG, and pathway functional annotation analyses were completed. We analysed the differentially expressed proteins in the negative control and interaction samples according to the protein data identified by MS and screened the interacting proteins of EspE3 in CTE.

### Statistical analysis

All experiments were performed three times. The statistical significance between groups was determined using the t test with GraphPad Prism v.8 (San Diego, CA, USA). *, *P* < 0.05; **, *P* < 0.01; and ***, *P* < 0.001 were considered statistically significant. ns: no significant difference.

## Results

### *espE3* gene locus map and protein analysis

**Figure 1:**
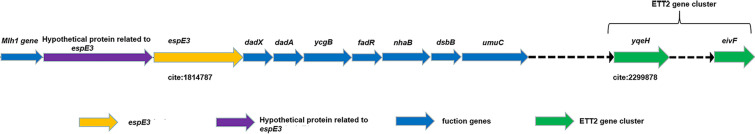
***espE3* gene locus map.** Blue represents some other functional genes in the APEC81 genome; Purple represents a predicted gene encoding a hypothetical protein related to *espE3*; Yellow represents *espE3* gene, and green represents genes encoded by ETT2 gene cluster.

When we analysed the genome sequence of AE81, we found that there was a gene sequence encoding a protein similar to the *Salmonella* SlrP effector [17, 18], which we called EspE3. *espE3* is located approximately 485091 bp before the ETT2 gene cluster in the AE81 genome, as shown in Figure 1. The gene sequence upstream of the *espE3* gene is suspected to encode a protein that is similar to the structural complex of SlrP and its host target protein.

In BLAST nucleotide searches, we found that *espE3* and its encoded protein were not named or annotated in *E. coli*. This gene has more than 99% homology with the compared gene sequence. It occurs in various serotypes of *E. coli*, including O157, O148, O18, and O78, and it occurs in both animal- and human-derived *E. coli* (Table 2). We predicted the promoter position and amino acid sequence of the gene. The predicted results showed that the gene encoded a protein with 423 amino acid residues (Additional file 2A). The three-dimensional structure and functional domain prediction results showed that the protein had a leucine repeat enrichment domain (Additional files 2B, C). The amino acid sequence alignment results showed that EspE3 and SlrP have a similarity greater than 46% (Additional file 2D). We calculated the distribution of EspE3 in 107 clinical isolates and found that the percent of strains with the gene was 31.78% (34/107).

**Table 2:** **Homologous gene information of** ***espE3-lacking E3.***

Description	Source	Homologous	NCBI accession
*Escherichia coli* TUM18780 DNA, complete genome	Homo sapiens	99.85%	AP023197.1
*Escherichia coli* O157:H16 strain Santai, complete genome	Duck	99.85%	CP007592.1
*Escherichia coli* strain E-T84-1 chromosome, complete genome	Homo sapiens	99.85%	CP090272.1
*Escherichia coli* strain 88COLEC chromosome, complete genome	Homo sapiens	99.85%	CP070906.1
*Escherichia coli* THO-010 DNA, complete genome	Homo sapiens	99.85%	AP022540.1
*Escherichia coli* strain RHB11-C15 chromosome, complete genome	Unknown	99.78%	CP057888.1
*Escherichia coli* strain B185/O148:H10/fimH562/3577 (ST Warwick) genome assembly, chromosome: 1	Unknown	99.27%	OU349838.1
*Escherichia coli* strain FDAARGOS_943 chromosome, complete genome	Unknown	99.27%	OU349838.1
*Escherichia coli* O18ac:H14 strain 873.10 chromosome, complete genome	Animal	99.47%	CP061754.1
*Escherichia coli* strain ECNB21-M121 chromosome, complete genome	Animal	99.34%	CP083585.1

### ETT2 genes regulate the transcription of EspE3 during the logarithmic growth phase

**Figure 2:**
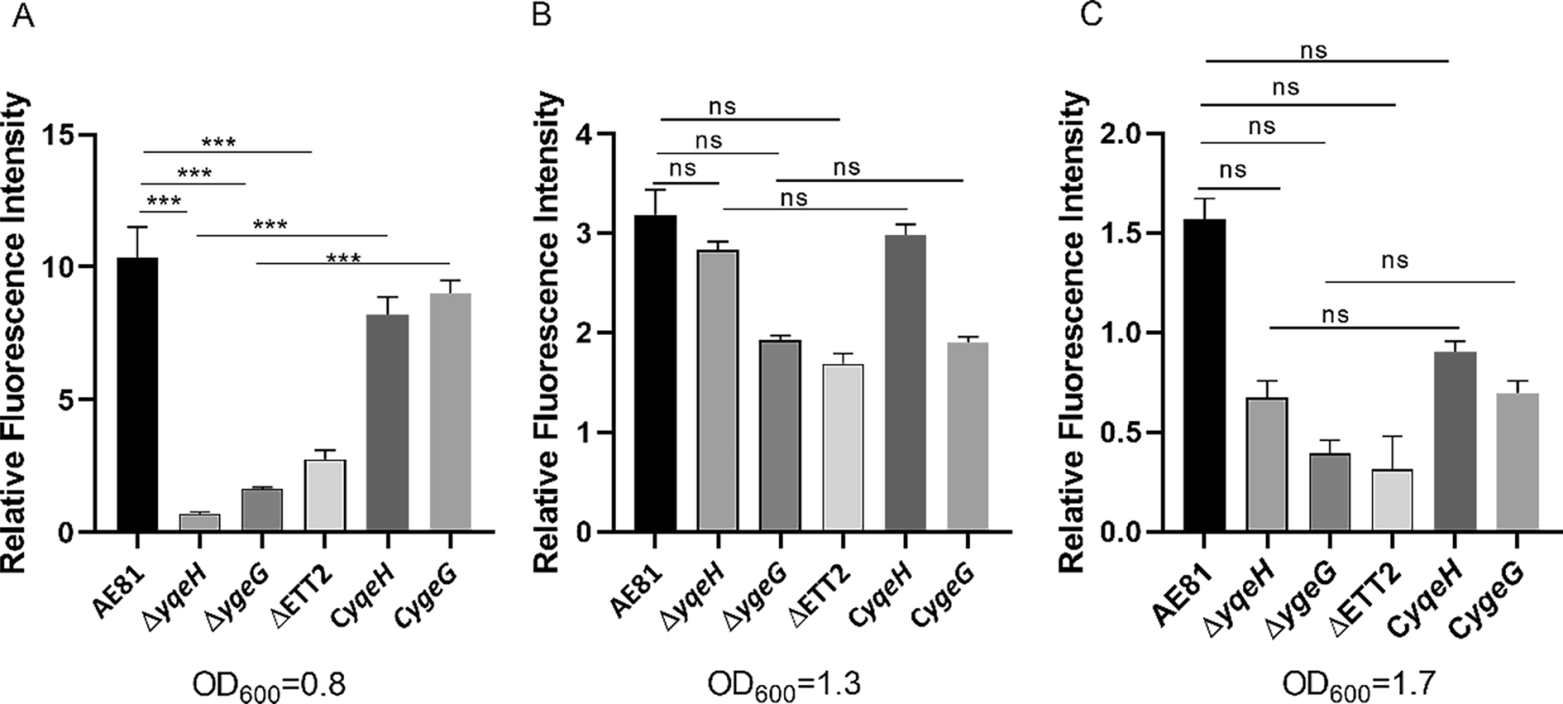
**Transcription level of *espE3* in different growth phases of each gene mutant.** We used APEC16S gene as an internal reference to detect the transcription level of *espE3* of different strains. **A** In the lag phase, the transcription level of *espE3* in the deletion strains of *yqeH*, *ygeG* and ETT2 were decreased compared with that of the wild strains (AE81), and the complementary strains had obvious recovery. **B**, **C** In the logarithmic phase and stationary phase, there was no significant difference in the transcription level of *espE3* between the three gene deletion strains and the wild strains, and the same was true for the complementary strains.

The results of qPCR detection showed that in the logarithmic growth phase of APEC (OD_600_ = 0.8), Δ*ygeG*, Δ*yqeH* and ΔETT2 showed a significant downregulation of *espE3* transcription (*P* ≤ 0.05), as shown in Figure 2A. In other growth phases (OD_600_ = 1.3, 1.7), the transcription level of *espE3* was not significantly decreased (*P* > 0.05) due to ETT2, *ygeG* and *yqeH* deletion (*P* > 0.05) (Figures 2B, C).

### Evaluation of the biological characteristics of pathogenicity and virulence of ***espE3***

**Figure 3:**
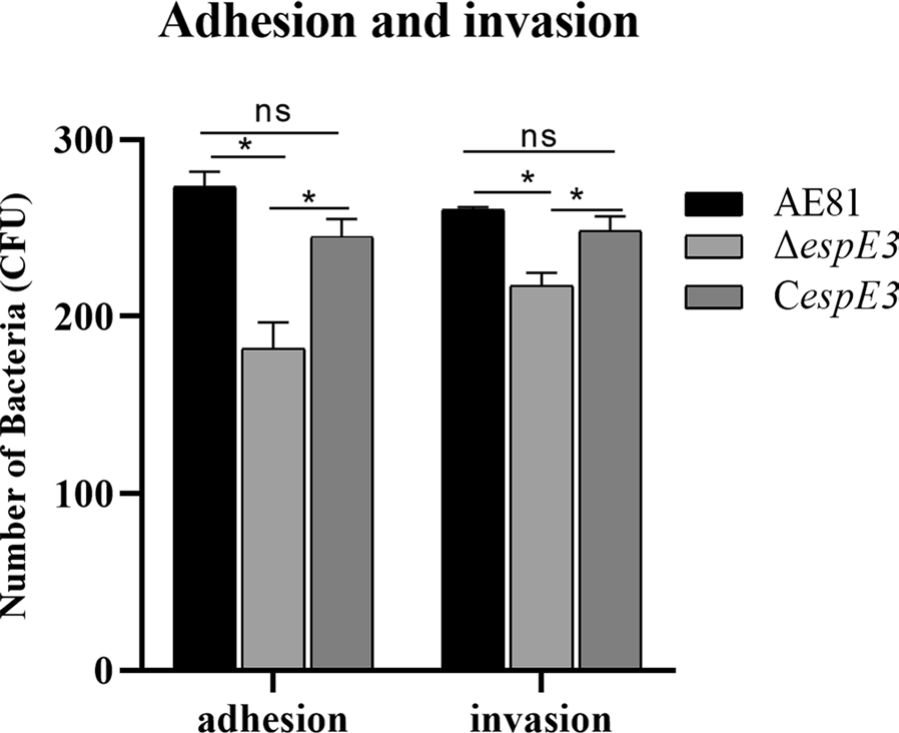
**Adhesion and invasion.** We diluted the samples after bacterial infection, cultured them in LB solid medium and counted the number of colonies. The results showed that the adhesion and invasion ability of the *espE3* deletion strain were significantly decreased, and the complementary strain had a significant recovery.

We verified that Δ*espE3* and C*espE3* were successfully constructed (Additional files 3A, B). AE81, Δ*espE3* and C*espE3* were used to measure the biological characteristics of pathogenicity. The results of the growth curve test showed that deletion of the *espE3* gene did not affect the growth ability of APEC (*P* > 0.05) (Additional file 4A). The same results were obtained in the motility test (*P* > 0.05) (Additional file 4B), the biofilm formation test (*P* > 0.05) (Additional file 4C) and the serum sensitivity test (*P* > 0.05) (Additional file 4D). However, the detection results of cell adhesion and invasion ability showed that Δ*espE3* significantly reduced the ability to adhere to CTE (*P* ≤ 0.05) and invade (*P* ≤ 0.05), as shown in Figure 3.

**Figure 4:**
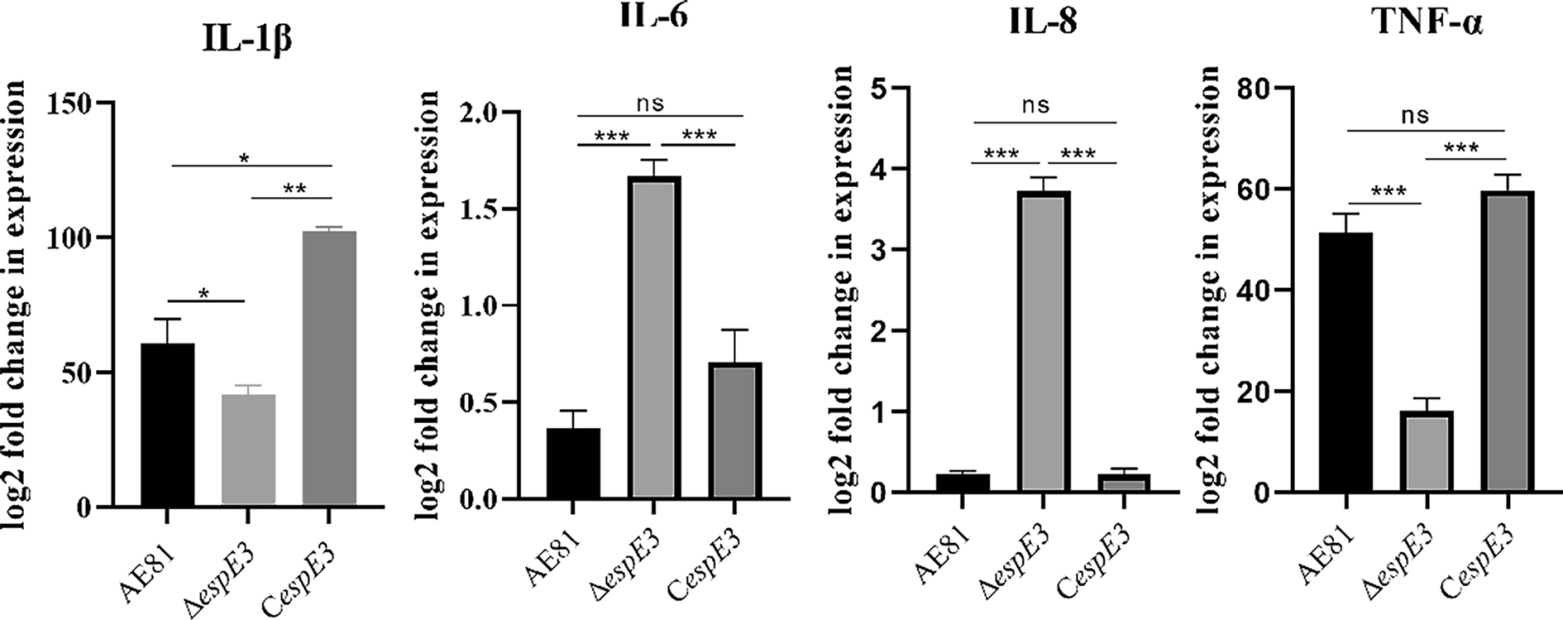
**Transcription levels of inflammatory cytokines.** We carried out RT-qPCR on bacterial infected cell samples, and the transcription level of IL-1β and TNF-α decreased significantly after the cells were infected by the *espE3* deletion strain, and the transcription level of the complementary strain recovered. The transcription levels of IL-6 and IL-8 were significantly increased after the infection of the cells with the *espE3* deletion strain, and the transcription levels of the complementary strain were restored.

To determine whether EspE3 stimulates the production of inflammatory factors, we used the three strains AE81, ∆*espE3* and C*espE3 *to infect CTE. The transcription level of inflammatory factors was measured by qRT‒PCR. The results showed that *espE3* significantly promoted IL-1β and TNF-α transcription in cells infected with CTE for 12 h (*P* ≤ 0.05) but significantly inhibited IL-6 (*P* ≤ 0.05) and IL-8 (*P* ≤ 0.05) transcription, as shown in Figure 4.

**Figure 5:**
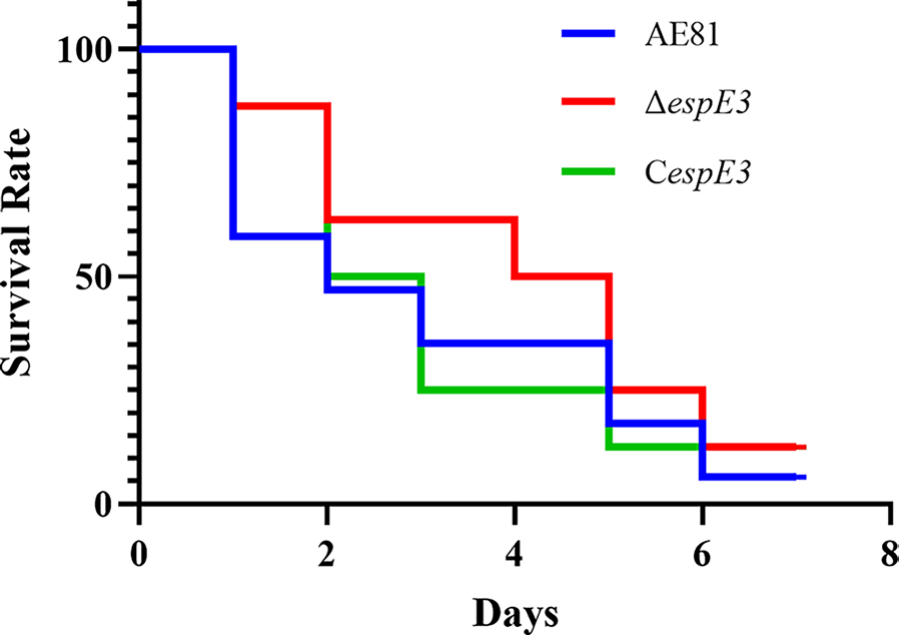
**Survival rate of chickens infected with AE81, Δ*espE3* and C*espE3.*** After infecting chicks with the *espE3* deletion strain, the complementary strain and the wild strain, we calculated the number of chicks surviving every day thereafter. On the first day, only 60% of chicks infected with wild strain survived, while 85% of chicks infected with *espE3* deletion strain survived, and 85% of chicks infected with complementary strain survived. There was a difference in the survival rate between chicks infected with the complementary strain and chicks infected with the *espE3* deletion strain on the second day. The survival rate of chicks infected with the *espE3* deletion strain within 7 days after infection were significantly higher than the other two strains.

To confirm whether *espE3* affects the pathogenicity of APEC, we performed a chicken challenge to determine survival rates and pathological changes in chick organs. The survival rates of chickens given AE81 and C*espE3* were lower than that of those given Δ*espE3*, as shown in Figure 5, which indicated that Δ*espE3* has lower pathogenicity.

**Figure 6:**
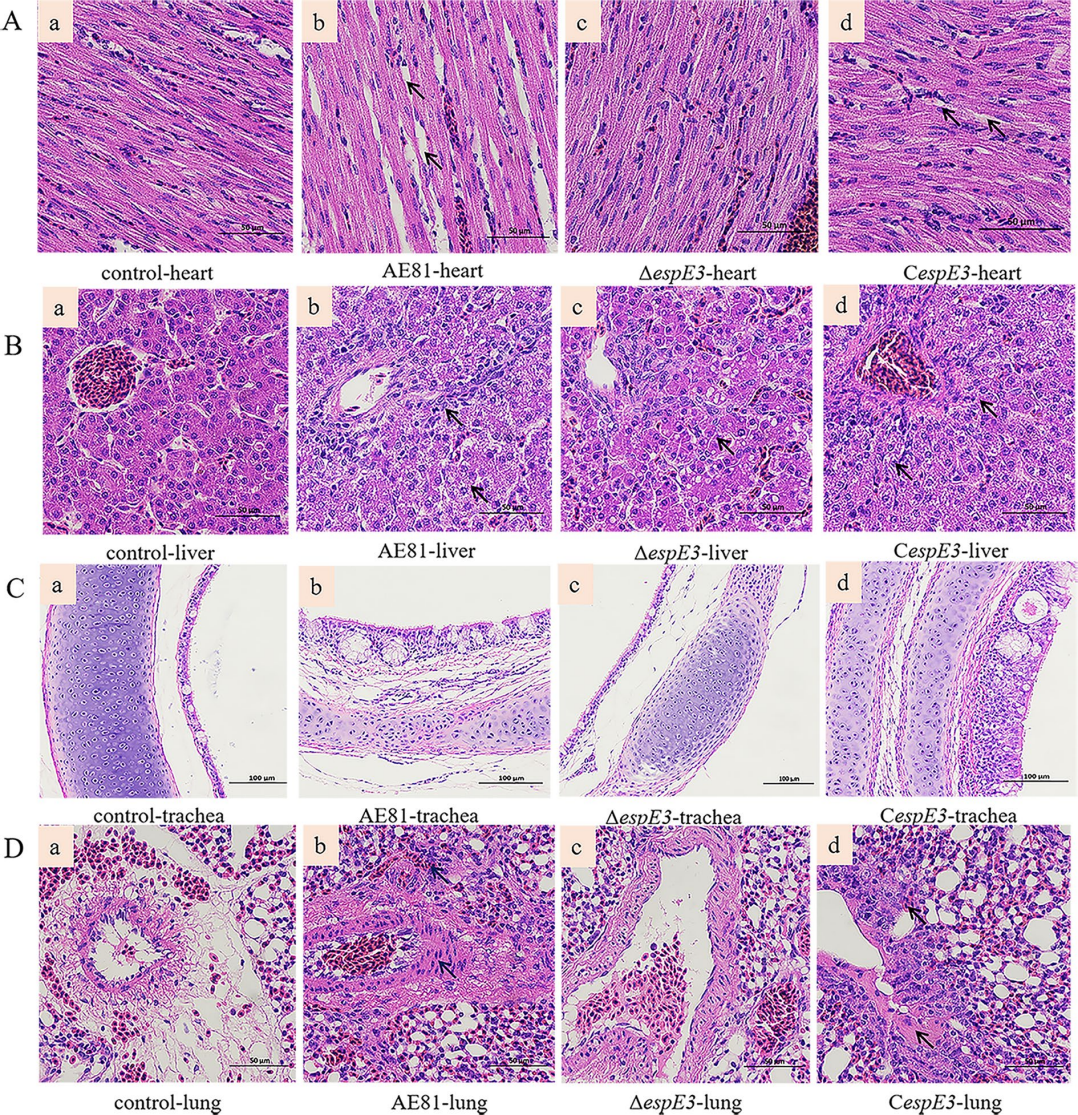
**Effect of EspE3 on the pathogenicity of APEC.** **A** In the heart, the loss of *espE3* alleviates the necrosis, roughness and deformation of myocardial fibers. **B** In the liver, the loss of *espE3* alleviated the degeneration of hepatocytes and blurred cell edges. **C** In the trachea, the loss of *espE3* alleviates the degeneration and proliferation of tracheal epithelial cells, the congestion of tracheal mucosa and the exudation of mucus. **D** In the lung, the loss of *espE3* leads to a significant reduction in inflammatory and proliferative lesions of cells.

Furthermore, under a light microscope with a 200 × field of view, pathological tissue sections showed that EspE3 enhanced the pathogenicity of APEC, as shown in Figure 6. In the challenged heart tissue, deletion of EspE3 led to a significant reduction in the APEC heart toxicity (Figure 6A). In liver tissues, deletion of EspE3 led to a significant reduction in APEC damage and the denaturation of visceral cells (Figure 6B). In the challenged tracheal tissues, deletion of EspE3 led to a significant reduction in the damage and inflammatory and proliferative lesions in tracheal mucosa and cells induced by APEC (Figure 6C). In challenged lung tissue, deletion of EspE3 led to a significant reduction in the inflammatory and proliferative lesions induced by APEC (Figure 6D).

### EspE3 can be secreted out of APEC

We used the T7-F/R primer to confirm that the BL21-pET-32a-EspE3 expression vector was successfully constructed (Additional files 5A, B). His-EspE3 could be expressed in large amounts in the supernatant and inclusion body, and the size of the protein was approximately 66.85 kDa (Additional file 5C). The purified protein (Additional file 5D) was detected by Western blotting (Additional file 5E). The purified His-EspE3 protein could be detected by polyclonal antibody serum, and the EspE3 protein secreted by the AE81 wild strain was simultaneously detected, whereas no protein bands were detected in the secreted proteins of the ΔETT2 strain (Additional file 5F).

### EspE3-interacting proteins in CTE were screened by GST pull-down

Identified with the pGEX-F/R primer, the constructed BL21-pGEX-6P-1-EspE3 amplification band was 1376 bp in length, and the empty body amplification band was 76 bp in length, indicating that the vector was successfully constructed; purified bands of approximately 76 kDa in length were obtained. Finally, Western blotting results verified that the GST-EspE3 protein was purified (Additional file 6).

**Figure 7:**
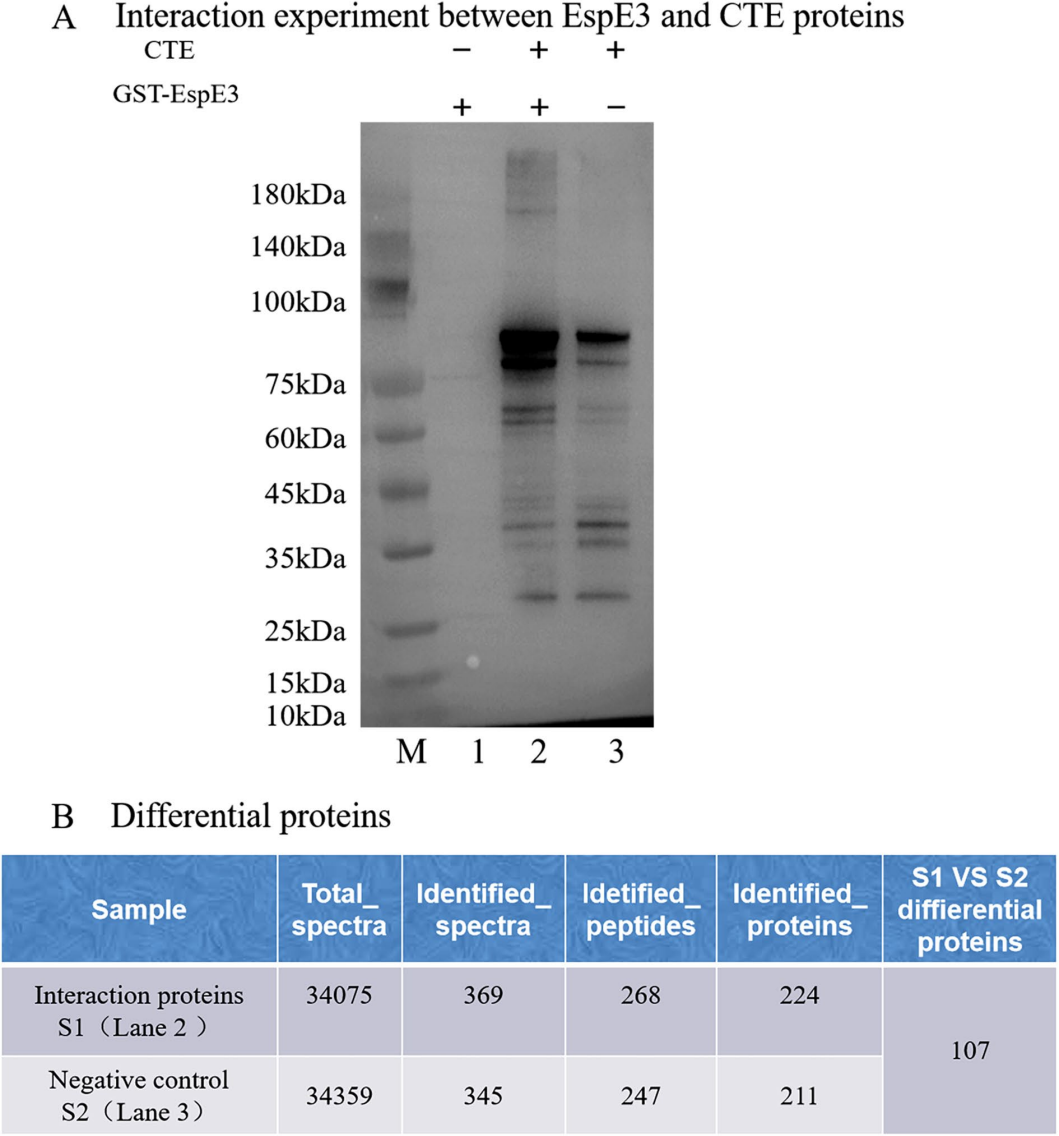
**Screening of proteins that interact with EspE3 in CTE.** **A** Interaction experiment between EspE3 and CTE proteins. 1: GST-EspE3 was used as bait, and PBS was used as prey. The interaction combined with eluent was used as a negative control. 2. GST-EspE3 was used as bait, and CTE protein was used as prey. 3: PBS was used as bait, and CTE protein was used as prey. **B** The total number of interacting proteins and the number of differential proteins were identified by mass spectrometry and sequencing.

Using GST-EspE3 as bait and CTE proteins as prey, the interaction target proteins were screened, and multiple differential protein bands were obtained, as shown in Figure 7A, with a total of 107 host interaction proteins screened (Figure 7B).

Using the UniProt *Gallus gallus* (chicken) database as the reference protein library, the pathways of the selected interacting proteins were analysed, and multiple innate immune response pathways, including the PI3K-Akt signalling pathway, the RNA transport pathway, the mTOR signalling pathway, the proteoglycan synthesis pathway, the axon-directed regeneration pathway, the cell cycle pathway, the DNA replication pathway, the PPAR signalling pathway, the endocytosis pathway, the viral infection pathway, and the animal mitotic phagocytosis pathway were identified (Table 3).

## Discussion

In this study, a gene located before the ETT2 gene cluster was found, which encoded a protein that is similar to SlrP [17, 18], with multiple β-turns and a leucine-rich repeat (LRR) domain in the middle of the protein model, and there is an α screw at the N-terminus. This gene/protein was named *espE3*/EspE3 in this study. The LRR domain is commonly found in effectors of the IpaH class of T3SS, such as SspH 1 [19], SspH 2 [20], YopM [21] and IpaH 9.8 [22]. The function of IpaH class effectors is closely related to the function of E3 ubiquitin ligases. IpaH effectors recruit host substrates for ubiquitination through the LRR domain [23]. We speculate that the LRR domains of EspE3 may have similar functions, recruiting host-specific substrates to play a pathogenic role in the host infection stage. The α screw at the N-terminus of EspE3 was the same as that of IpaH3, and there was a CxxxD (x represents any amino acid residue) motif in EspE3 at 266–270 aa, which is similar to the CxD motif in IpaH3. The CxD motif is conserved, located in a loop region, and harbours a cysteine residue that catalyses ubiquitin transfer through a transthiolation reaction. We speculate that EspE3 may also have a similar E3 ubiquitin ligase function to IpaH3. At the same time, through sequence comparison using the NCBI BLAST tool, it was found that the gene occurs widely in many serotypes of *E. coli* and in both animal-derived and human-derived *E. coli*.

To determine the transcriptional relationship of *espE3* and ETT2, three different ETT2 deletion strains were selected for qRT‒PCR. The transcription of various effectors is regulated by transcriptional regulators of the secretion system, so we explored whether YqeH could regulate the transcription level of *espE3*. YqeH can directly regulate the transcription of flagellin and quorum sensing and play an important role in regulating the pathogenicity of APEC [9]. We indeed found that the transcription of *espE3* was positively regulated by YqeH, suggesting that YqeH also promotes pathogenicity by regulating the transcription of *espE3*. The chaperone proteins of the secretion system can assist effector proteins in folding correctly and transporting them to the substrate platform before secretion [24]; they can also control the secretion timing of effector proteins to achieve the best infection effect [25]. In this study, the ETT2 chaperone YgeG positively regulated *espE3* transcription. Therefore, we speculated that YqeH and YgeG may accelerate the pathogenic process of APEC during infection by regulating *espE3* transcription. In ΔETT2, *espE3* transcription was not affected, which may be due to the possibility of there being other factors in ETT2 that regulate *espE3* transcription and the possibility of various regulatory effects affecting and offsetting each other; however, this idea needs to be further explored.

We confirmed that EspE3 did not affect the growth, motility, biofilm formation ability or sensitivity of APEC to serum, while EspE3 promoted APEC adhesion and the invasion of host cells. The enhancement of adhesion and invasion generally depends on the increase in flagella and fimbriae and the enhancement of their functions [26]. EspE3 does not affect the characteristics dominated by flagella and fimbriae, such as motility and biofilm formation, indicating that the mechanism of promoting adhesion and invasion is not related to flagella and fimbriae enhancement. EspE3 may alter some antibacterial properties of host cells (such as host cell surface protein structure or inflammatory response), thus significantly enhancing the adhesion and invasion ability of APEC into host cells.

We found that EspE3 significantly promoted the transcription of TNF-α and significantly inhibited the transcription of IL-6 and IL-8 in CTE-infected cells for 12 h. IL-6, IL-8 and TNF-α are important inflammatory factors [27, 28], and the change in their transcription level indicates that EspE3 is involved in the interaction between APEC and host cells. The E3 ubiquitinase effectors of most T3SSs, after entering the host cell, act on the ubiquitinated receptor substrates of the innate immune pathway of the host cell, hinder the ubiquitination reaction of the immune pathway, and then play a role in inhibiting or stimulating the innate immune pathway of the host [22, 29, 30]. Therefore, we speculate that EspE3 can regulate the activation of inflammatory pathways in CTE, help APEC resist killing by innate immune mechanisms, and promote its colonization and proliferation in host target cells.

Through animal experiments, we found that EspE3 increased the mortality rate of APEC infection and enhanced the pathological changes related to organ inflammation. The trachea is the first target organ of APEC in natural infection, and it shows pathological changes when EspE3 is produced/expressed. APEC infection leads to clear morphological changes, degeneration and proliferation in tracheal mucosal cells, resulting in a vacuole. Similar to the functions of other T3SS effector factors [31], this may be due to the interaction between EspE3 and some proteins in epithelial cells, resulting in damage to the physical barrier of epithelial cells, which facilitates the adhesion and invasion of APEC into host cells. The lung is the second site of infection connected to the trachea, resulting in tissue proliferation and inflammation. Moreover, the liver and heart also showed inflammatory symptoms mediated by EspE3, indicating that the presence of EspE3 stimulated the inflammatory response of the host, reflecting the important contribution of EspE3 to the pathogenicity of APEC.

We detected EspE3 using our lab-made antibody and found that EspE3 was secreted by ETT2 and played a pathogenic role. To further explore the specific receptors by which EspE3 interacts with CTE during infection, we screened 107 host-interacting proteins involved in a variety of innate immune response pathways. Although these interactions need to be further validated and their mechanisms explored, we list some of the interacting proteins here. Because other interacting molecules of EspE3 have yet to be verified and further studied, complete data on the interacting proteins will not be provided in this study. If there is a scientific need, readers can apply to the corresponding author.

We report the protein EspE3 secreted by APEC-ETT2 and explore the transcriptional regulation between ETT2 and *espE3*. Our results demonstrated that EspE3 enhances the pathogenicity of APEC to host cells, alters the transcription levels of host inflammatory cytokines after infection of host epithelial cells, and induces severe inflammatory lesions in the heart, liver, trachea, and lung of the host. The 107 interacting proteins of EspE3, including eIF4H, SEM3A, EPHB3, STAG2, SERPINF2, MCM5, and UBB, were screened out. In conclusion, our study shows that EspE3 is a secreted protein that plays significant roles in the pathogenicity of APEC in chicks (Table 3).

**Table 3:** **Some interacting proteins in chicken tracheal epithelial cells.**

Protein ID	Gene name	Pathway
tr|A0A1L1S0V6|A0A1L1S0V6_CHICK	eIF4H	PI3K-Akt signalling pathway
		RNA transport
		mTOR signalling pathway
		Proteoglycans in cancer
SEM3A_CHICK	SEM3A	Axon guidance
sp|Q07498|EPHB3_CHICK	EPHB3	Axon guidance
tr|E1BSU3|E1BSU3_CHICK	STAG2	Cell cycle
tr|F1NAR5|F1NAR5_CHICK	SERPINF2	Complement and coagulation cascade
tr|A0A1D5NW89|A0A1D5NW89_CHICK	MCM5	Cell cycle
		DNA replication
sp|P0CG62|UBB_CHICK	UBB	PPAR signalling pathway
		Endocytosis
		Herpesvirus infection
		Animal mitotic phagocytosis

### Supplementary Information

**Additional file 1. Primers used in this study.** This table contains the primer sequences used in this study.

**Additional file 2. Protein model analysis of EspE3 in the AE81 genome.** This figure is the result of software analysis of the protein model encoded by the gene sequence of AE81-*espE3*.

**Additional file 3. Δ*espE3* and C*espE3* were constructed.** This figure shows the identification of strains Δ*espE3* and C*espE3* by PCR.

**Additional file 4. Evaluation of biological characteristics of pathogenicity.** Biological characteristic experiments were conducted on AE81, Δ*espE3* and C*espE3*, including growth curve determination, motility, biofilm formation ability, and serum resistance, without significant differences seen in the results.

**Additional file 5. Expression, purification and identification of the fusion protein EspE3.** Identify the expression, purification, and secretion of EspE3.

**Additional file 6. Prokaryotic expression of the GST-EspE3 protein.** Identify the expression, purification of GST-EspE3.

## Data Availability

The data that support the findings of this study are available on request from the corresponding author, [KQ]，upon reasonable request.
