# Supplementary figures and images for: EspE3 plays a role in the pathogenicity of avian pathogenic Escherichia coli
Source: Vet Res. 2023 Aug 29;54:70. doi: 10.1186/s13567-023-01202-9 (PMC10463865; doi:10.1186/s13567-023-01202-9)

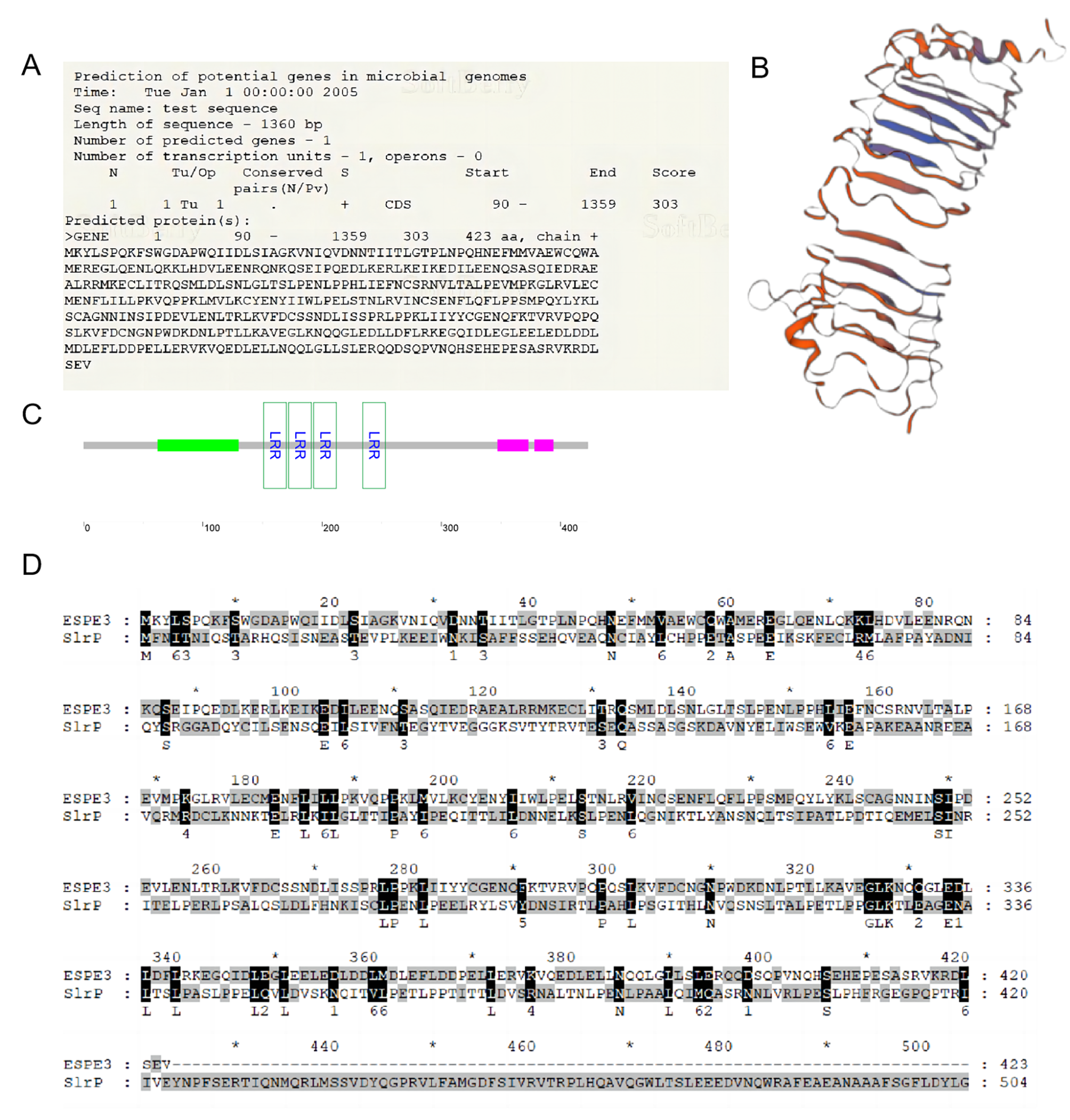

Supplement: Supplementary file 2 — Additional file 2. Protein model analysis of EspE3 in the AE81 genome. This figure is the result of software analysis of the protein model encoded by the gene sequence of AE81-espE3. [file 13567_2023_1202_MOESM2_ESM.png]

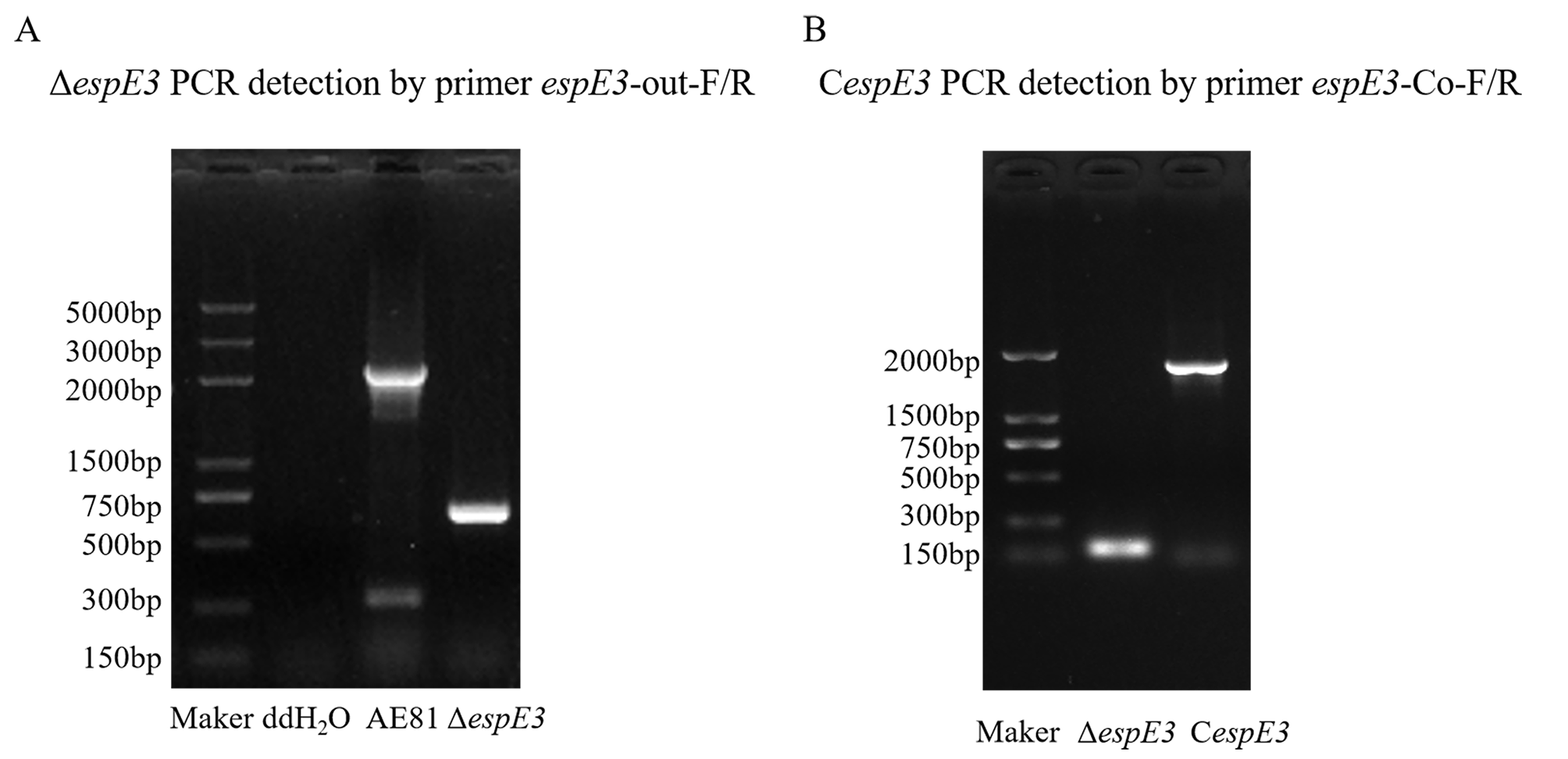

Supplement: Supplementary file 3 — Additional file 3. ΔespE3 and CespE3 were constructed. This figure shows the identification of strains ΔespE3 and CespE3 by PCR. [file 13567_2023_1202_MOESM3_ESM.png]

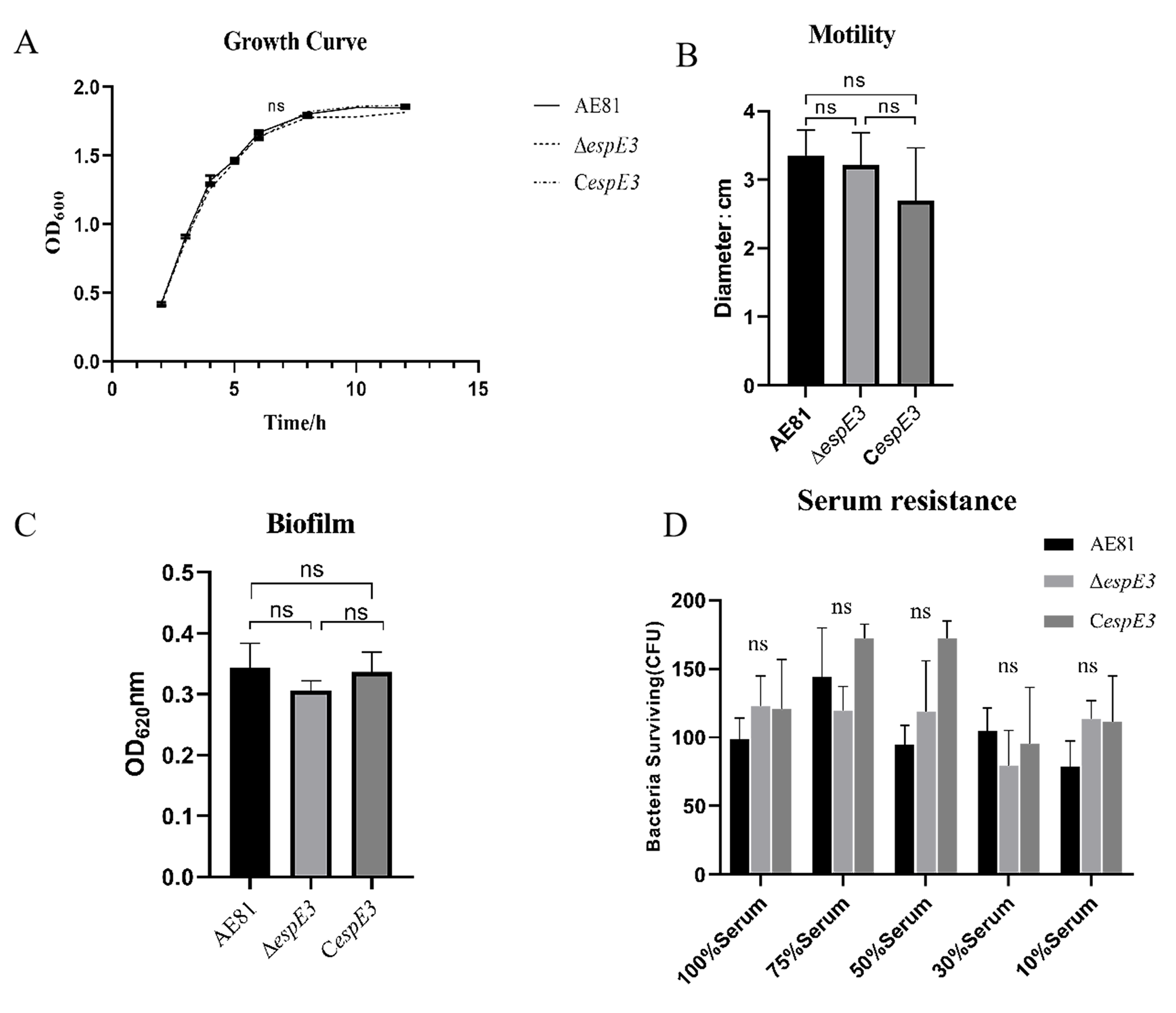

Supplement: Supplementary file 4 — Additional file 4. Evaluation of biological characteristics of pathogenicity. Biological characteristic experiments were conducted on AE81, ΔespE3 and CespE3, including growth curve determination, motility, biofilm formation ability, and serum resistance, without significant differences seen in the results. [file 13567_2023_1202_MOESM4_ESM.png]

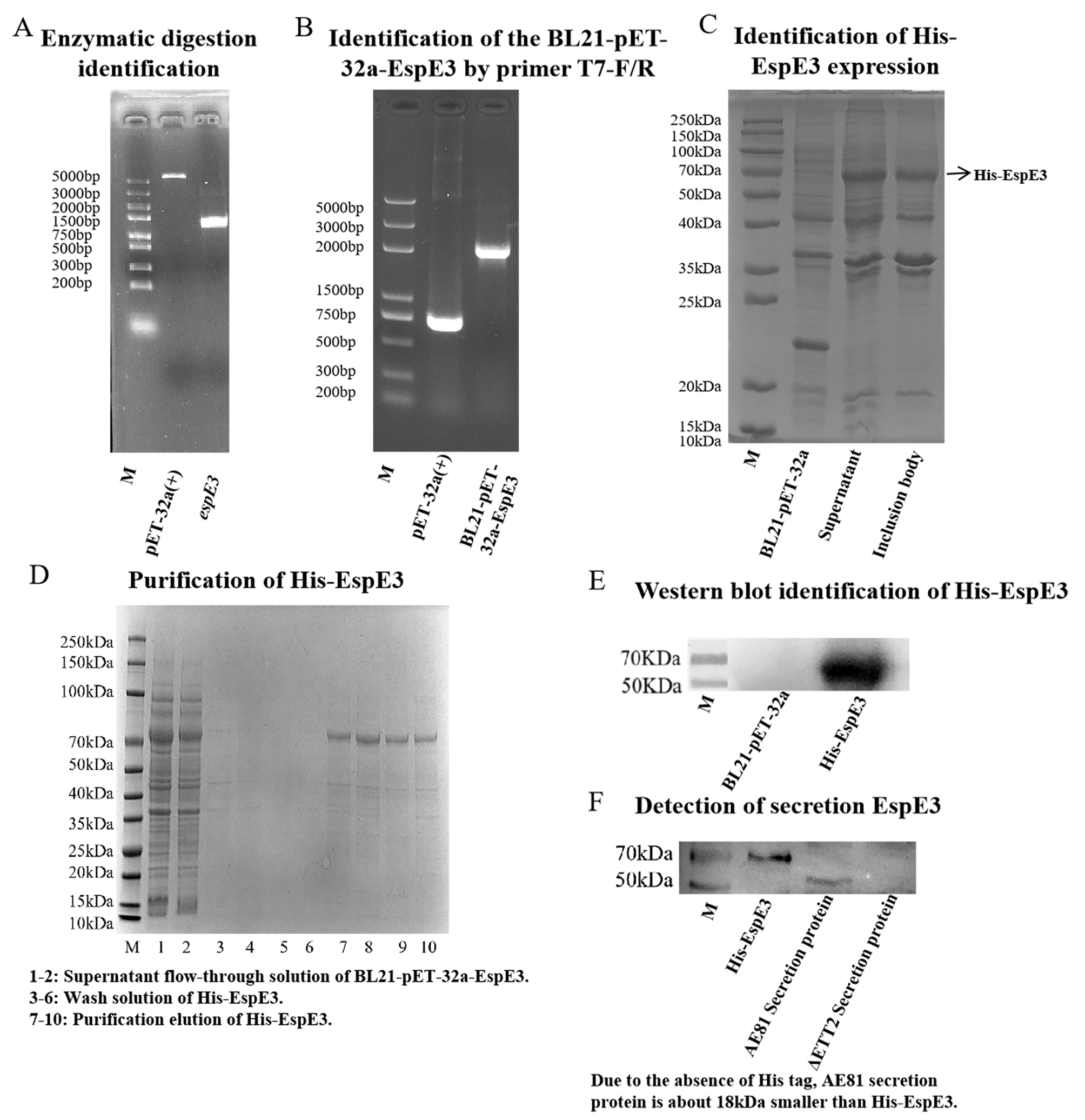

Supplement: Supplementary file 5 — Additional file 5. Expression, purification and identification of the fusion protein EspE3. Identify the expression, purification, and secretion of EspE3. [file 13567_2023_1202_MOESM5_ESM.png]

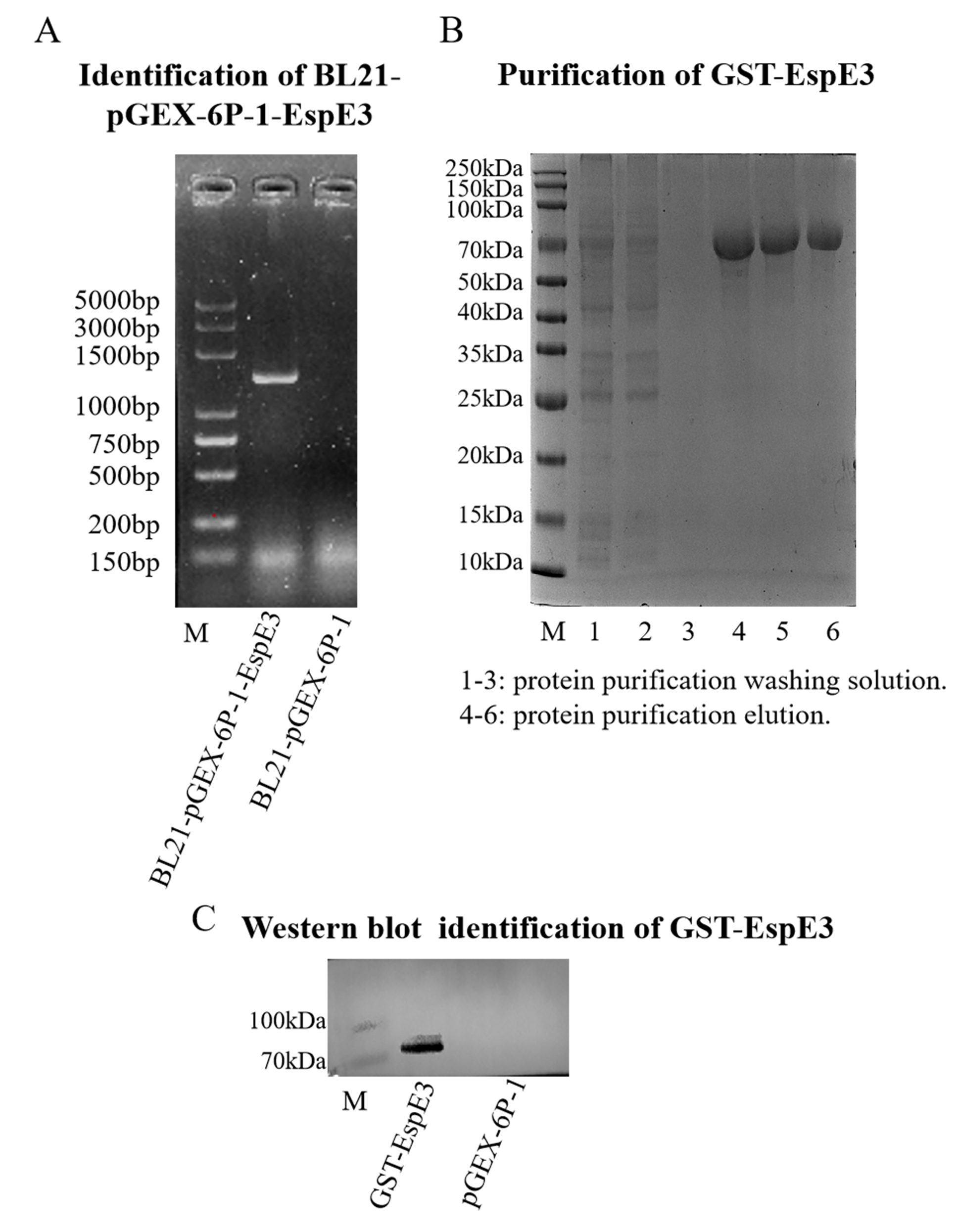

Supplement: Supplementary file 6 — Additional file 6. Prokaryotic expression of the GST-EspE3 protein. Identify the expression, purification of GST-EspE3. [file 13567_2023_1202_MOESM6_ESM.png]
